# Independent risk factors for clinically significant acute poisoning in children presenting to the emergency department: a 4-year cohort study of 2,345 cases

**DOI:** 10.3389/fped.2026.1881926

**Published:** 2026-07-08

**Authors:** Zhai Zhao, Xingsi Liang, Xiao Wang, Weili Guo, Wenjin Geng

**Affiliations:** Emergency Department, Children's Hospital of Hebei Province, Hebei Clinical Research Center for Children's Health and Diseases, Shijiazhuang City, China

**Keywords:** blood purification, epidemiology, multiple organ dysfunction syndrome, organ dysfunction, pediatric poisoning, poisoning severity score, psychotropic drugs, risk factors

## Abstract

**Background:**

Acute poisoning is a major pediatric emergency department (ED) presentation, yet evidence-based tools for early risk stratification at triage remain limited. This study aimed to characterize epidemiological trends and identify independent risk factors for clinically significant poisoning (Poisoning Severity Score [PSS] ≥ 2) in children.

**Methods:**

We conducted a retrospective cohort study of 2,345 children (<18 years) with acute poisoning (2021–2024). Patients were stratified into mild (PSS 0–1) and clinically significant (moderate-to-severe) groups. Multiple imputation handled missing data. Multivariable logistic regression identified independent risk factors. Model discrimination and calibration were assessed using the Area Under the Receiver Operating Characteristic curve (AUC-ROC) and Hosmer-Lemeshow test. Patient-centered outcomes (acute organ dysfunctions) were systematically analyzed.

**Results:**

Over 4 years, the poisoning spectrum shifted from agricultural chemicals toward psychotropic drugs (18.5%–31.2%). Clinically significant poisoning occurred in 422 patients (18.0%). Independent risk factors for moderate-to-severe outcomes were: age >12 years (aOR 2.34), intentional self-harm (aOR 3.12), pre-hospital delay >6 h (aOR 2.78), multiple-agent exposure (aOR 2.15), exposure to agricultural herbicides (aOR 5.45), and psychotropic drugs (aOR 3.86). The model achieved an outstanding AUC-ROC of 0.892 (95% CI: 0.864–0.920). Clinically significant poisoning (PSS ≥ 2) was strongly associated with patient-centered organ injury, including acute respiratory failure (26.5% vs. 0.1%, *P* < 0.001), toxic encephalopathy (22.5% vs. 0.0%, *P* < 0.001), and Multiple Organ Dysfunction Syndrome (MODS, 12.8% vs. 0.0%, *P* < 0.001) compared to mild cases. These cases required PICU admission (71.1%) and blood purification (44.1%) significantly more often. Mortality was 1.2% (28/2,345), occurring exclusively in the PSS ≥ 2 group.

**Conclusion:**

Readily identifiable risk factors—age >12 years, self-harm, delay >6 h, multiple-agent exposure, and exposure to herbicides or psychotropics—independently predict clinically significant pediatric poisoning. The PSS ≥ 2 classification correlates tightly with acute organ dysfunction and Multiple Organ Dysfunction Syndrome (MODS).

## 1 Introduction

### 1.1 Background

Acute pediatric poisoning remains a formidable global public health challenge and is recognized as one of the leading causes of non-traumatic morbidity, mortality, and disability among children and adolescents presenting to emergency departments (EDs) worldwide (1). The World Health Organization (WHO) has continuously highlighted the disproportionate burden of unintentional toxic exposures in early childhood, alongside an alarming global surge in intentional self-poisoning among adolescents (2). In recent decades, rapid urbanization, socio-economic transitions, and the widespread availability of synthetic compounds have profoundly altered the epidemiological landscape of pediatric poisoning (3, 4). Historically, the pediatric poisoning spectrum in developing regions was heavily dominated by agricultural chemicals, particularly organophosphates and highly toxic herbicides such as paraquat and diquat (5, 6). However, the contemporary poisoning spectrum is undergoing a significant paradigm shift. Recent multicenter epidemiological investigations have documented a marked transition towards pharmaceutical agents—particularly psychotropic drugs such as antidepressants, sedatives, and antipsychotics—and complex household chemical products (7). This evolution necessitates a continuous reassessment of clinical characteristics, as the pathophysiological mechanisms, clinical trajectories, and requisite medical interventions differ drastically between traditional agricultural chemical exposures and modern pharmaceutical overdoses (8).

### 1.2 Knowledge gap

Despite the critical importance of continuously monitoring these epidemiological shifts, existing literature on pediatric acute poisoning demonstrates several substantial limitations. Firstly, a significant proportion of published retrospective cohorts are limited by small sample sizes (typically fewer than 500 cases), which insufficiently capture the rare but life-threatening manifestations of clinically significant poisoning (1, 9). Secondly, there is a notable scarcity of recent, large-scale data encompassing the post-COVID-19 pandemic era (2021 onwards). The psychological aftermath of the pandemic has arguably exacerbated mental health crises among adolescents, potentially driving an unprecedented increase in intentional psychotropic drug overdoses (10), yet this trend lacks robust quantification in recent high-volume cohorts. Most importantly, previous studies have frequently treated pediatric poisoning as a homogenous entity, failing to strictly stratify patients based on standardized severity indices. The indiscriminate merging of “mild/accidental ingestions” (typically characterized by a Poisoning Severity Score [PSS] of 0 to 1) with “moderate-to-severe cases” (PSS ≥ 2) obscures the identification of true predictive variables (11). The lack of rigorous multivariable regression models designed specifically to isolate independent risk factors for clinically significant poisoning—adjusting for crucial confounders such as pre-hospital admission delay, multiple-agent exposures, specific toxicant subclasses, and patient developmental stages—leaves a critical gap in evidence-based emergency triage protocols (12).

### 1.3 Objective

To bridge these critical knowledge gaps, we designed a comprehensive, large-scale, single-center retrospective cohort study spanning four consecutive years (January 2021 to December 2024). The primary objective of this study is to elucidate the contemporary epidemiological evolution and temporal trends of the pediatric acute poisoning spectrum. The secondary and core objective is to identify and quantify the independent clinical and demographic risk factors driving clinically significant poisoning (defined as PSS ≥ 2). Furthermore, we sought to substantiate the clinical validity of our severity stratification by examining its association with objective patient-centered outcomes, including specific acute organ dysfunctions and Multiple Organ Dysfunction Syndrome (MODS). By strictly stratifying patients according to severity and toxicant etiology, we aim to provide robust, high-quality evidence that may inform future downstream efforts to optimize ED triage systems, facilitate early risk stratification, and guide the rational allocation of advanced critical care resources (13). Furthermore, to ensure the highest standards of observational research reporting, this manuscript was drafted in strict accordance with the Strengthening the Reporting of Observational Studies in Epidemiology (STROBE) guidelines established by the EQUATOR Network (14).

## 2 Methods

### 2.1 Study design and setting

This was a large-scale, single-center, retrospective observational cohort study conducted at a premier tertiary pediatric medical center, which serves as the central hub for pediatric emergency and critical care in the region. The study period encompassed four consecutive years, from January 1, 2021, to December 31, 2024. The study protocol was reviewed and strictly approved by the Institutional Review Board (IRB) and the independent Ethics Committee of the hospital. Given the retrospective nature of the study, which utilized de-identified and anonymized electronic medical record (EMR) data without any direct patient intervention, the requirement for obtaining written informed consent was formally waived by the Ethics Committee. Furthermore, the design, implementation, and reporting of this study adhered strictly to the Strengthening the Reporting of Observational Studies in Epidemiology (STROBE) guidelines to ensure maximal methodological transparency and rigor (14).

### 2.2 Study population

The target population comprised pediatric patients who presented to the emergency department with a primary diagnosis of acute poisoning, a frequent cause of pediatric emergency visits (10). The inclusion criteria were defined as follows: (1) chronological age strictly less than 18 years at the time of presentation; (2) a definite and documented clinical history of acute exposure to single or multiple toxic substances (including ingestion, inhalation, dermal absorption, or injection); and (3) admission occurring within the specified study timeframe (2021–2024). To minimize potential confounding factors and ensure the homogeneity of the acute toxicological cohort, the exclusion criteria were rigorously established as: (1) patients who were dead on arrival (DOA) or suffered cardiac arrest prior to ED admission; (2) cases with severe data deficiencies (defined as missing more than 20% of core demographic or clinical variables); (3) chronic poisoning or prolonged environmental exposures (e.g., chronic lead poisoning); (4) food poisoning (e.g., bacterial gastroenteritis, botulism), allergic reactions, or adverse drug events occurring at prescribed therapeutic dosages; and (5) envenomation by venomous animals (e.g., snakebites, bee stings), as their pathophysiological mechanisms and treatment paradigms diverge significantly from chemical poisonings.

### 2.3 Data collection and Variable definitions

Clinical data were systematically extracted from the hospital's integrated EMR system using a standardized electronic case report form (eCRF) by two independent trained clinical researchers. Any discrepancies during data extraction were resolved through consensus or consultation with a senior pediatric toxicologist. The independent variables evaluated in this study included extensive demographic characteristics, exposure profiles, and clinical timelines. Demographic variables incorporated age and gender. Age was further stratified into four developmental stages for clinical relevance: infants and toddlers (<3 years), preschoolers (3–6 years), school-aged children (7–12 years), and adolescents (> 12 years). Toxicant categories were meticulously classified into six major groups: (1) Agricultural chemicals (sub-classified into organophosphates, paraquat/diquat, and others); (2) Psychotropic medications (e.g., benzodiazepines, SSRIs, antipsychotics); (3) Over-the-counter (OTC) and non-psychotropic prescription drugs (e.g., acetaminophen, ibuprofen, cardiovascular agents); (4) Household chemicals and daily necessities (e.g., detergents, cosmetics, bleach); (5) Biological agents and poisonous plants (e.g., toxic mushrooms); and (6) Miscellaneous/Unknown agents. In instances of polypharmacy or multiple-agent exposures, cases were categorized based on the primary (dominant) toxicant responsible for the predominant clinical toxidrome, as evaluated by the treating clinical toxicologist. We also specifically recorded the presence of “multiple-agent exposure (poly-intoxication)” as a distinct binary variable. The route of exposure was categorized based on the intent as either “unintentional ingestion/accidental” or “intentional self-harm/suicide attempt.” The time from ingestion to ED admission (pre-hospital delay) was recorded as a continuous variable and subsequently categorized into <2, 2–6, and >6 h based on the typical therapeutic window for gastric decontamination procedures. In addition, to account for potential confounding before tertiary-care admission, we extracted information on documented pre-referral management. Because detailed records on inter-hospital transfer status, exact timing and dose of decontamination, antidotal therapy, lipid emulsion therapy, and other toxin-specific treatments were not consistently available in the retrospective EMR, pre-hospital intervention was analyzed as a binary variable indicating whether preliminary gastrointestinal decontamination, such as gastric lavage and/or activated charcoal, had been performed at another facility before arrival at our ED.

### 2.4 Outcome measures and poisoning severity score

The severity of clinical manifestations was quantified utilizing the standardized Poisoning Severity Score (PSS), which categorizes poisoning into five grades based on objective clinical signs, symptoms, and laboratory findings: PSS 0 (None: no symptoms or signs), PSS 1 (Minor: mild, transient, and spontaneously resolving symptoms), PSS 2 (Moderate: pronounced or prolonged symptoms), PSS 3 (Severe: severe or life-threatening symptoms), and PSS 4 (Fatal: death) (15). For the purpose of this analysis, the primary clinical endpoint was the occurrence of “clinically significant poisoning,” defined *a priori* as a PSS ≥ 2 (moderate, severe, or fatal poisoning), consistent with recent pediatric clinical toxicology research in which PSS ≥ 2 was used as a severity criterion alongside clinically meaningful outcomes such as pediatric intensive care admission, coma, and respiratory depression (16). Importantly, to accurately capture the true clinical trajectory, the PSS was assigned based on the peak (worst) clinical severity documented during the patient's entire hospital stay, rather than solely relying on the initial ED presentation. Patients with a PSS of 0 or 1 were aggregated into the “mild poisoning” cohort. Secondary outcome measures included the rate of Pediatric Intensive Care Unit (PICU) admission, the requirement for advanced extracorporeal treatments specifically blood purification modalities (such as continuous venovenous hemodiafiltration [CVVHDF] or hemoperfusion [HP]), and the overall all-cause in-hospital mortality rate. Furthermore, to substantiate the clinical relevance of the PSS stratification with hard clinical endpoints, we evaluated major acute organ dysfunctions and systemic complications as patient-centered outcomes, including acute respiratory failure, acute kidney injury (AKI), acute liver injury, myocardial injury, toxic encephalopathy, shock, and Multiple Organ Dysfunction Syndrome (MODS).

### 2.5 Statistical analysis

All statistical analyses were executed using R software (version 4.2.1, R Foundation for Statistical Computing, Vienna, Austria) and IBM SPSS Statistics for Windows (version 26.0, IBM Corp., Armonk, NY, USA). The threshold for statistical significance was set at a two-tailed *P*-value of < 0.05. Missing data occurred in pre-hospital admission delay (3.1%), toxicant category/subclass (0.5%), and clinical parameters used for PSS calculation (1.1%). Missingness was assumed to be Missing at Random (MAR). To avoid selection bias and preserve statistical power, Multiple Imputation by Chained Equations (MICE) was performed using the *mice* package in R to generate five imputed datasets, utilizing predictive mean matching for continuous variables and logistic regression for categorical variables. Statistical analyses were run on each imputed dataset and subsequently pooled using Rubin's rules. Continuous variables were assessed for normality using the Kolmogorov–Smirnov test. Normally distributed data were expressed as means with standard deviations (SD) and compared using the Student's t-test, whereas non-normally distributed data were presented as medians with interquartile ranges (IQR) and analyzed using the Mann–Whitney *U* test. Categorical variables were summarized as absolute frequencies and percentages, with group comparisons performed utilizing the Pearson Chi-square test or Fisher's exact test, as appropriate.

To identify the independent prognostic risk factors associated with clinically significant poisoning (PSS ≥ 2), candidate predictors were selected based on clinical relevance and a univariate significance threshold of *P* < 0.10. Variables including age group, gender, intent of exposure, pre-hospital admission delay, toxicant categories, multiple-agent exposure, and pre-hospital interventions met these criteria and were incorporated into the multivariable model. A backward stepwise elimination procedure (based on the likelihood ratio) was utilized to refine the model. The magnitude of association was expressed as adjusted odds ratios (aOR) accompanied by their corresponding 95% confidence intervals (CI). Prior to finalizing the regression model, collinearity diagnostics were rigorously performed; Variance Inflation Factor (VIF) values were calculated, and a VIF threshold of < 2.5 was utilized to exclude multicollinearity among the independent variables. Model discrimination was evaluated using the Area Under the Receiver Operating Characteristic curve (AUC-ROC) with 95% CIs. Model calibration was assessed via the Hosmer-Lemeshow goodness-of-fit test, where a *P*-value > 0.05 indicated optimal calibration. Internal validation was performed using 1,000 bootstrap resamples to compute the optimism-corrected AUC-ROC to evaluate the model's stability and generalizability.

## 3 Results

### 3.1 Study population and baseline characteristics

**Figure 1:**
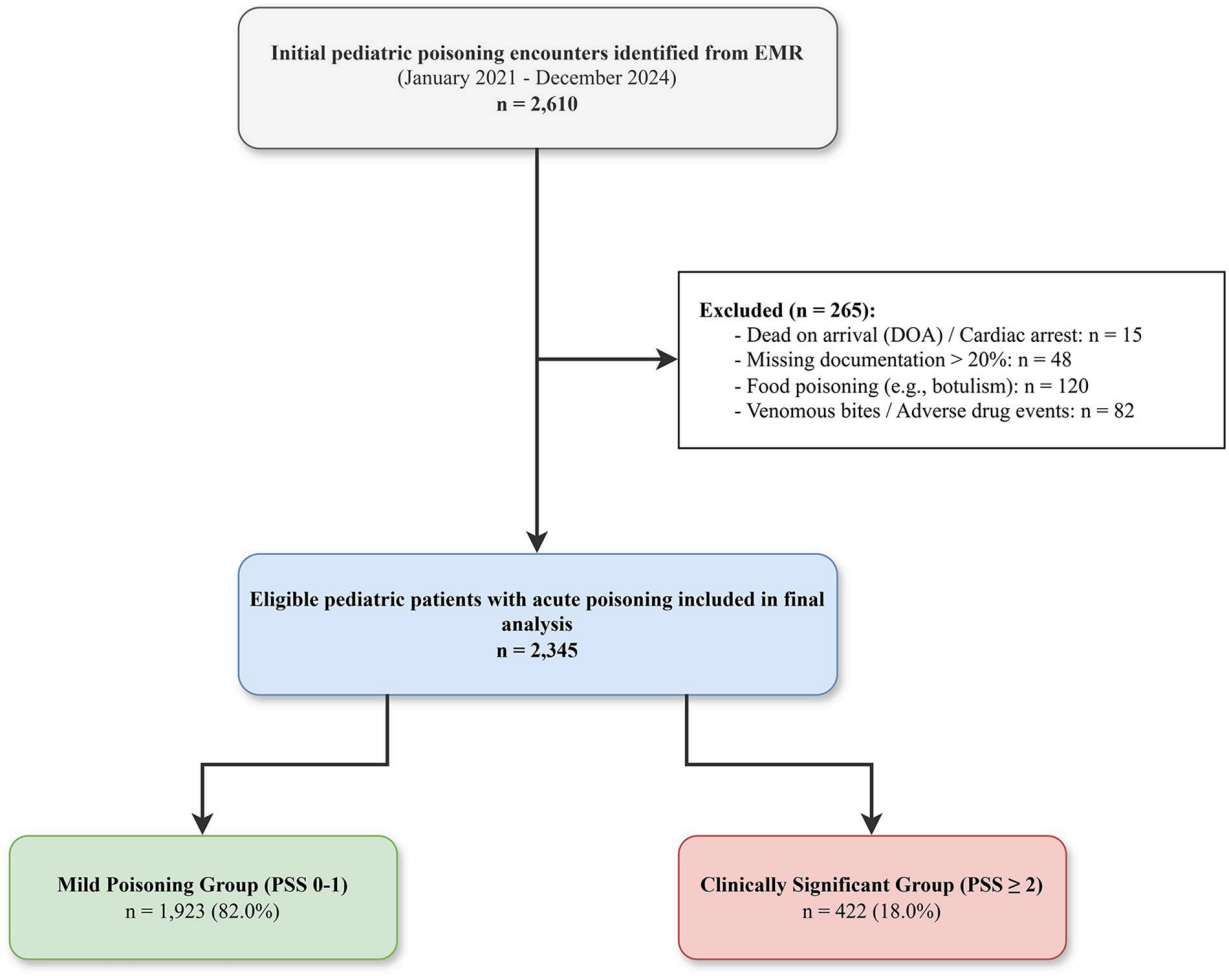
Patient inclusion flowchart. A detailed schema illustrating the step-by-step selection process of the pediatric poisoning cohort from January 2021 to December 2024. The flowchart delineates the initial number of queried electronic medical records, specific exclusion criteria (e.g., dead on arrival, missing data, food poisoning), and the final number of eligible patients included in the multivariable analysis.

During the four-year study period from January 2021 to December 2024, an initial total of 2,610 pediatric patient encounters with a preliminary diagnosis of acute poisoning were identified in the EMR system. Following the rigorous application of predefined exclusion criteria, 265 cases were excluded (including 15 cases of DOA, 48 cases with substantial missing documentation, 120 cases of suspected food poisoning, and 82 cases involving venomous bites or therapeutic adverse drug events). Consequently, a definitive cohort of 2,345 eligible patients was included in the final statistical analysis. The detailed patient selection process is graphically depicted in the study inclusion flowchart (Figure 1). Within the final cohort, the median age was 6.5 years (IQR: 3.2–13.8 years), comprising 1,215 males (51.8%) and 1,130 females (48.2%). Notably, multiple-agent exposure (poly-intoxication) was documented in 365 patients (15.6%), and 450 patients (19.2%) had received some form of pre-hospital intervention at a local facility prior to tertiary admission. The detailed baseline demographic and clinical characteristics of the overall study population are comprehensively summarized in Table 1.

**Table 1:** Baseline demographic and clinical characteristics of the overall pediatric poisoning cohort (2021–2024, *N* = 2,345).

Characteristics	Total patients (*N* = 2,345)	Percentage (%)
Age (years), median (IQR)	6.5 (3.2–13.8)	–
Age groups		
Infants and toddlers (<3 years)	492	21.0
Preschoolers (3–6 years)	635	27.1
School-aged (7–12 years)	408	17.4
Adolescents (>12 years)	810	34.5
Gender		
Male	1,215	51.8
Female	1,130	48.2
Intent of exposure		
Unintentional/Accidental	1,833	78.2
Intentional/Self-harm	512	21.8
Multiple-agent exposure		
Single agent	1,980	84.4
Multiple agents (Poly-intoxication)	365	15.6
Pre-hospital admission delay		
<2 h	1,210	51.6
2–6 h	593	25.3
>6 h	542	23.1
Pre-hospital intervention		
No	1,895	80.8
Yes (e.g., prior gastric decontamination)	450	19.2
Major toxicant categories		
Agricultural chemicals	352	15.0
Psychotropic drugs	586	25.0
OTC/Prescription drugs (Non-psycho)	469	20.0
Household chemicals	610	26.0
Biologicals/Plants	188	8.0
Miscellaneous/Unknown	140	6.0

Values are expressed as counts (*n*) and percentages (%) unless specified otherwise. IQR, interquartile range; OTC, over-the-counter. Age is presented as median (IQR) because of its highly skewed, non-normal distribution (as confirmed by the Kolmogorov–Smirnov test, *P* < 0.001). Multiple-agent exposure indicates poly-intoxication. Pre-hospital intervention refers to documented preliminary gastrointestinal decontamination, such as gastric lavage and/or activated charcoal, performed at another facility before ED arrival.

### 3.2 Evolution of poisoning Spectrum and epidemiological trends

**Figure 2:**
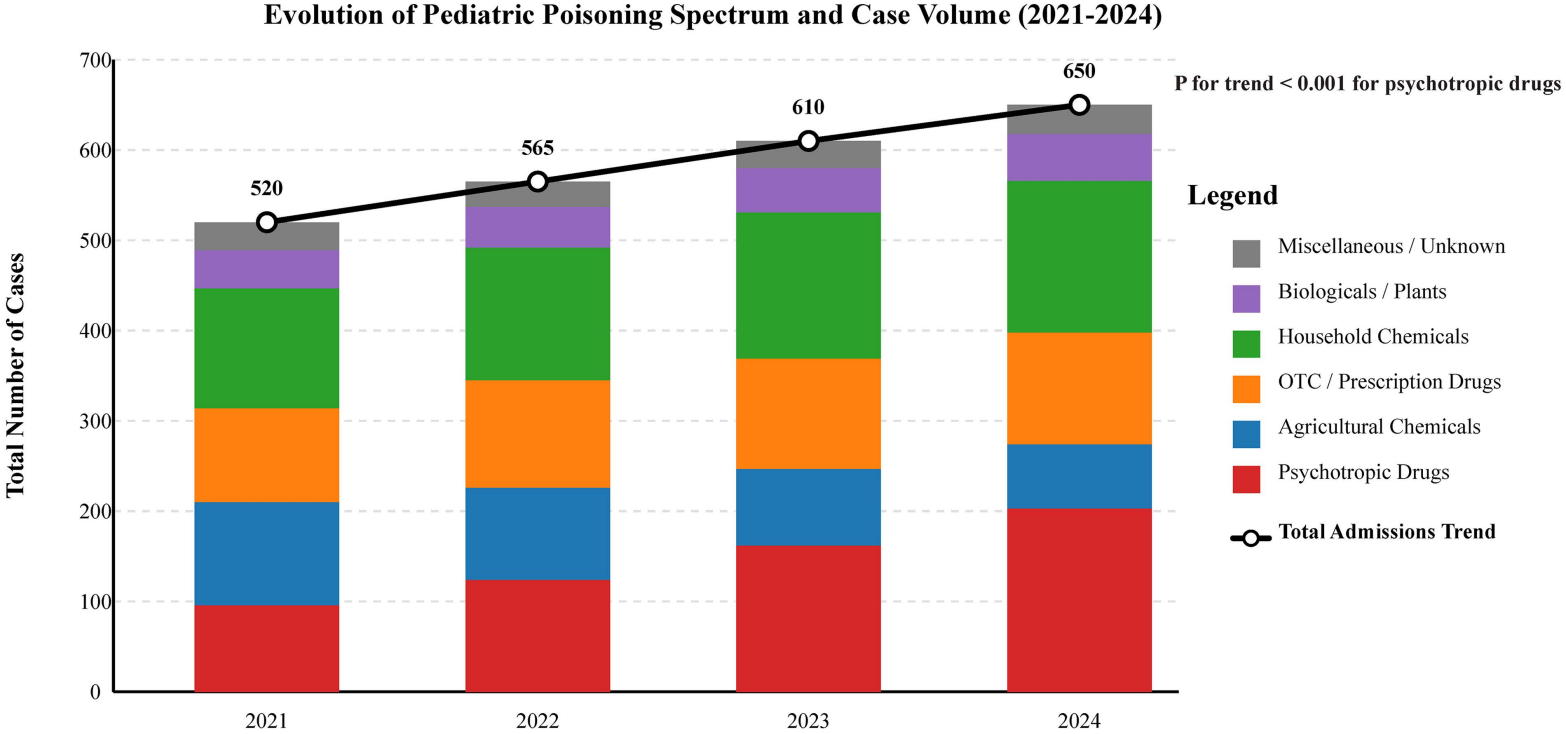
Evolution of the pediatric poisoning spectrum (2021–2024). A composite stacked bar chart and trend line. The stacked bars represent the proportional distribution of major toxicant categories (Agricultural Chemicals, Psychotropic Drugs, OTC/Prescription Drugs, Household Chemicals, Biologicals, and Others) across the four-year study period. The superimposed solid line depicts the absolute upward trend in total annual pediatric poisoning admissions to the emergency department. The increasing trend in psychotropic drug exposures from 18.5% in 2021 to 31.2% in 2024 was statistically significant by the Cochran-Armitage trend test (*P* < 0.001).

**Figure 3:**
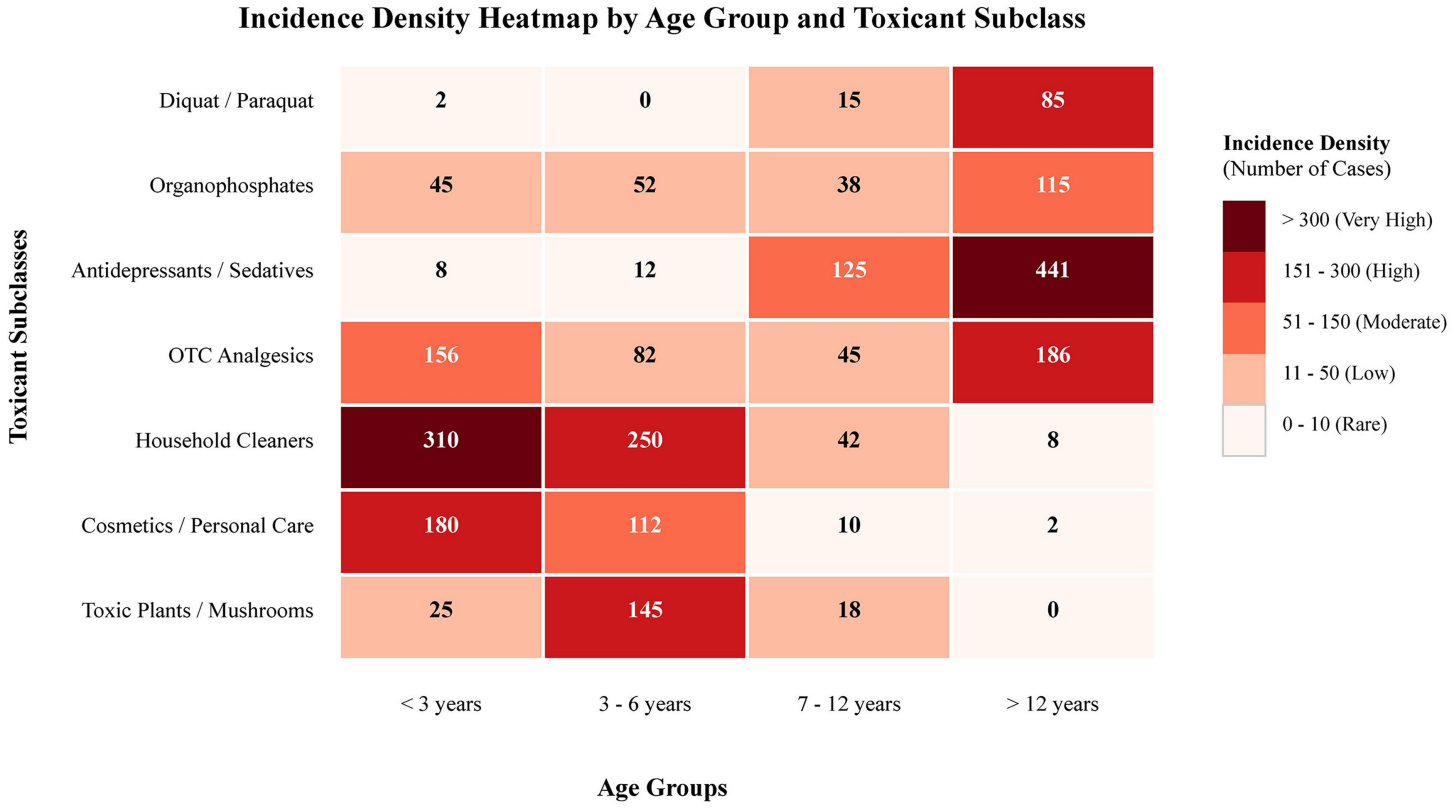
Incidence density heatmap by age group and toxicant subclass. A color-coded incidence heatmap visualizing the intersection of developmental age groups (*x*-axis: <3 years, 3–6 years, 7–12 years, >12 years) and specific toxicant subclasses (*y*-axis: diquat/paraquat, organophosphates, antidepressants, household cleaners, etc.). Deeper color intensities represent a higher incidence density of cases. The map highlights a distinct bimodal distribution: accidental ingestion of household products in preschoolers vs. intentional psychotropic self-harm in adolescents.

A temporal analysis of the four-year data revealed dynamic fluctuations in the total volume of admissions and a distinct paradigm shift in the toxicological spectrum. The annual case volume demonstrated a progressive upward trajectory: 520 cases in 2021, 565 in 2022, 610 in 2023, and a peak of 650 cases in 2024. The composition of the poisoning spectrum transformed significantly across the timeline (Figure 2). While exposure to traditional agricultural chemicals (such as organophosphates) experienced a marginal proportional decline, the incidence of poisonings involving pharmaceutical agents, particularly psychotropic drugs, surged dramatically from 18.5% in 2021 to 31.2% in 2024 (*P* < 0.001 for trend, Cochran-Armitage test). Furthermore, the distribution of poisoning incidents exhibited a striking bimodal pattern heavily dictated by age and intent, which is visually delineated in the incidence density heatmap (Figure 3). The primary peak occurred among infants and preschoolers (<6 years), characterized overwhelmingly by unintentional ingestions of household chemicals, over-the-counter medications, and toxic plants. Conversely, the secondary peak emerged among adolescents (>12 years), dominated predominantly by intentional self-harm and suicidal behaviors utilizing psychotropic medications (e.g., antidepressants and sedatives) and highly lethal agricultural herbicides (e.g., diquat and paraquat).

### 3.3 Clinical characteristics: comparison between mild and clinically significant cases

Based on the PSS stratification, 1,923 patients (82.0%) were categorized into the mild poisoning group (PSS 0–1), while 422 patients (18.0%) met the criteria for the clinically significant (moderate-to-severe) poisoning group (PSS ≥ 2). Univariate analysis revealed profound clinical discrepancies between the two cohorts (Table 2). Compared to the mild group, patients in the clinically significant group were significantly older (median age 14.2 years vs. 4.5 years, *P* < 0.001) and exhibited a higher proportion of females (58.5% vs. 45.9%, *P* < 0.001). The etiology of exposure diverged drastically: intentional self-harm constituted 65.4% of the PSS ≥ 2 cases, compared to only 12.3% in the mild cohort (*P* < 0.001). Pre-hospital presentation delay was also a critical differentiator; 45.3% of moderate-to-severe cases presented to the ED more than 6 h post-ingestion, in stark contrast to 18.2% in the mild group (*P* < 0.001). Additionally, multiple-agent exposure was significantly more prevalent in the clinically significant group compared to the mild group (37.9% vs. 10.7%, *P* < 0.001). Furthermore, exposures to agricultural chemicals and psychotropic drugs were overwhelmingly overrepresented in the PSS ≥ 2 cohort.

**Table 2:** Univariate analysis comparing clinical characteristics between mild (PSS 0-1) and clinically significant (PSS ≥ 2) poisoning groups.

Variables	Mild group (PSS 0-1) (*n* = 1,923)	Clinically significant group (PSS ≥ 2) (*n* = 422)	*P*-value
Age (years), median (IQR)	4.5 (2.8–9.1)	14.2 (11.5–16.8)	<0.001
Gender, *n* (%)			<0.001
Male	1,040 (54.1)	175 (41.5)	
Female	883 (45.9)	247 (58.5)	
Intent of exposure, *n* (%)			<0.001
Unintentional/Accidental	1,687 (87.7)	146 (34.6)	
Intentional/Self-harm	236 (12.3)	276 (65.4)	
Multiple-agent exposure, *n* (%)			<0.001
Single agent	1,718 (89.3)	262 (62.1)	
Multiple agents	205 (10.7)	160 (37.9)	
Pre-hospital delay, *n* (%)			<0.001
<2 h	1,120 (58.2)	90 (21.3)	
2–6 h	452 (23.5)	141 (33.4)	
>6 h	351 (18.2)	191 (45.3)	
Pre-hospital intervention, *n* (%)			<0.001
No	1,583 (82.3)	312 (73.9)	
Yes	340 (17.7)	110 (26.1)	
Toxicant categories, *n* (%)			<0.001
Agricultural chemicals	210 (10.9)	142 (33.6)	
Psychotropic drugs	355 (18.5)	231 (54.7)	
OTC/Household/Others	1,358 (70.6)	49 (11.6)	
Clinical interventions, *n* (%)			<0.001
PICU admission	15 (0.8)	300 (71.1)	
Blood purification (CRRT/HP)	0 (0.0)	186 (44.1)	
In-hospital mortality, *n* (%)	0 (0.0)	28 (6.6)	<0.001

Values are expressed as counts (*n*) and percentages (%) within each group. PSS, Poisoning Severity Score; PICU, pediatric intensive care unit; CRRT, continuous renal replacement therapy; HP, hemoperfusion; IQR, interquartile range; OTC, over-the-counter medications. *P*-values were calculated using the Pearson Chi-square test (or Fisher's exact test) for categorical variables and the Mann–Whitney *U* test for Age.

### 3.4 Independent risk factors for clinically significant poisoning

**Figure 4:**
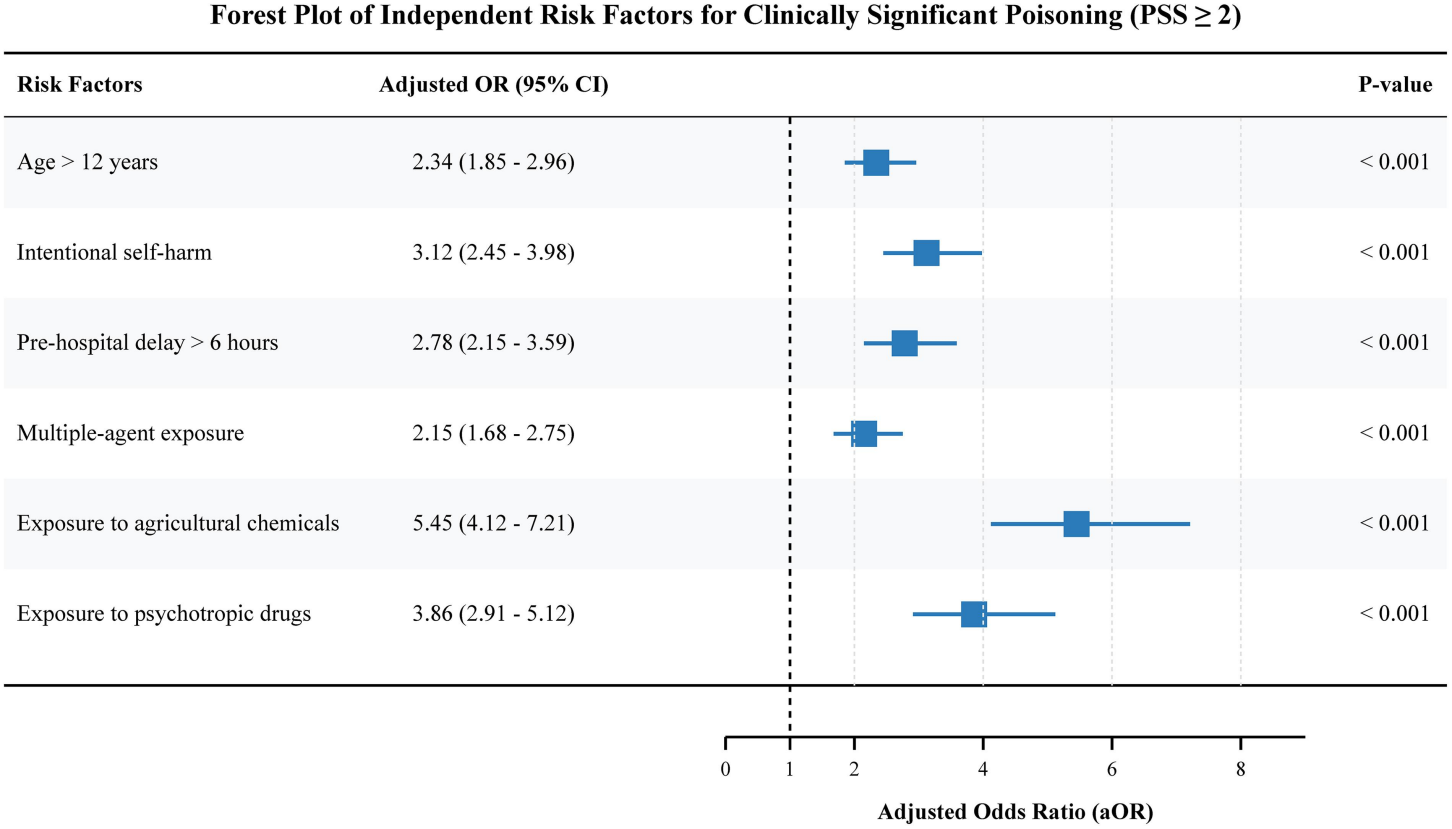
Forest plot of independent risk factors for clinically significant poisoning (PSS ≥ 2). A forest plot graphically summarizing the results of the multivariable logistic regression analysis. Adjusted odds ratios (aOR) and their corresponding 95% confidence intervals (CI) are presented for variables significantly associated with the development of clinically significant poisoning, including the newly added multiple-agent exposure predictor. The vertical dashed line represents an odds ratio of 1.0 (no effect). Solid squares represent the point estimates, with horizontal lines indicating the 95% CIs.

To isolate the definitive drivers of moderate-to-severe outcomes, variables demonstrating significance in the univariate analysis were incorporated into the multivariable logistic regression model. Collinearity diagnostics confirmed the absence of significant multicollinearity among the predictors, with all Variance Inflation Factors (VIF) falling well below 2.5 (specifically: age group, VIF = 1.34; intent of exposure, VIF = 1.48; pre-hospital delay, VIF = 1.12; exposure to agricultural chemicals, VIF = 1.25; exposure to psychotropic drugs, VIF = 1.31; multiple-agent exposure, VIF = 1.18; and pre-hospital intervention, VIF = 1.09). As comprehensively visualized in the Forest Plot (Figure 4), several variables emerged as robust independent risk factors for the development of clinically significant poisoning (PSS ≥ 2). Age > 12 years independently doubled the risk (adjusted Odds Ratio [aOR] 2.34, 95% CI: 1.85–2.96, *P* < 0.001). Intentional self-harm was an exceptionally strong predictor (aOR 3.12, 95% CI: 2.45–3.98, *P* < 0.001). Additionally, a pre-hospital admission delay exceeding 6 h significantly escalated the likelihood of adverse outcomes (aOR 2.78, 95% CI: 2.15–3.59, *P* < 0.001). Moreover, multiple-agent exposure was confirmed as a significant independent risk factor (aOR 2.15, 95% CI: 1.68–2.75, *P* < 0.001). Among toxicant categories, exposure to agricultural chemicals, particularly high-lethality herbicides (aOR 5.45, 95% CI: 4.12–7.21, *P* < 0.001), and psychotropic drugs (aOR 3.86, 95% CI: 2.91–5.12, *P* < 0.001) were confirmed as the most critical toxicological determinants driving clinical deterioration. Pre-hospital intervention was not independently associated with PSS ≥ 2 after adjustment (*P* = 0.14); however, this finding should not be interpreted as evidence of no treatment benefit, given the possibility of confounding by indication and incomplete documentation of pre-referral management.

The final multivariable model demonstrated excellent discriminative capacity, with an Area Under the Receiver Operating Characteristic curve (AUC-ROC) of 0.892 (95% CI: 0.864–0.920). Calibration of the model was confirmed by the Hosmer-Lemeshow test (*χ*^2^ = 5.72, *P* = 0.68), which indicated no significant discrepancy between predicted and observed outcomes. Internal validation via 1,000 bootstrap resamples yielded an optimism-corrected AUC-ROC of 0.885, proving the outstanding stability and generalization of our risk prediction model.

### 3.5 Clinical outcomes and advanced interventions

**Figure 5:**
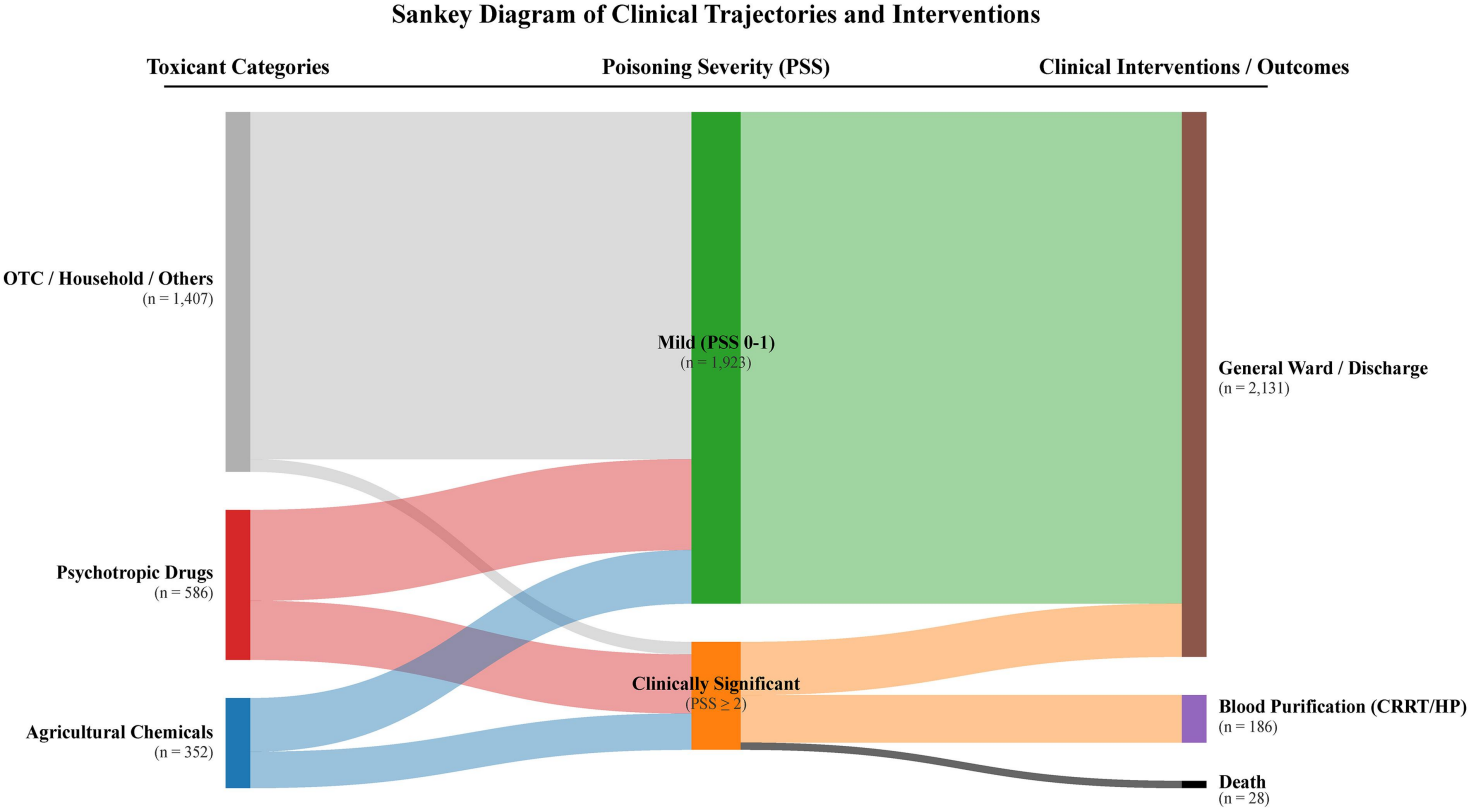
Sankey diagram of clinical trajectories and outcomes. A flow diagram tracking patient progression from initial toxicant exposure through clinical severity to ultimate medical outcomes. The left axis delineates the major toxicant classes (e.g., Psychotropic vs. Agricultural); the middle axis categorizes the Poisoning Severity Score (Mild vs. Clinically Significant); and the right axis displays major clinical pathways, including general ward/discharge, blood purification (CRRT/HP), and death. The thickness of the bands is directly proportional to the volume of patients following that specific clinical trajectory.

The severity of poisoning directly dictated the intensity of clinical interventions and the ultimate prognosis. Among the entire cohort, 315 patients (13.4%) necessitated admission to the PICU. Advanced extracorporeal blood purification, predominantly hemoperfusion (HP) or continuous renal replacement therapy (CRRT), was rapidly initiated in 186 cases (7.9%). The overall all-cause mortality rate for the cohort was 1.2% (*n* = 28), with all fatalities exclusively occurring within the clinically significant (PSS ≥ 2) group. The clinical trajectories—mapping toxicant exposure categories through severity stages to major clinical pathways (general ward/discharge, blood purification, or death)—are presented in the Sankey diagram (Figure 5). This visualization illustrates a distinct clinical divergence: clinically significant cases induced by psychotropic drugs frequently progressed to require aggressive blood purification but ultimately survived, whereas severe ingestions of specific agricultural chemicals (notably paraquat and diquat), despite maximal interventional efforts including CRRT, accounted for the overwhelming majority of the mortality burden.

### 3.6 Organ dysfunction and clinical complications

To further evaluate patient-centered outcomes, we analyzed the incidence of specific acute organ dysfunctions and systemic complications between the mild and clinically significant poisoning groups (Table 3). Patients in the clinically significant poisoning group (PSS ≥ 2) experienced significantly higher rates of acute respiratory failure (26.5% vs. 0.1%, *P* < 0.001), toxic encephalopathy (22.5% vs. 0.0%, *P* < 0.001), acute liver injury (19.9% vs. 0.8%, *P* < 0.001), myocardial injury (18.0% vs. 0.5%, *P* < 0.001), acute kidney injury (11.4% vs. 0.2%, *P* < 0.001), and shock (8.3% vs. 0.1%, *P* < 0.001). Notably, Multiple Organ Dysfunction Syndrome (MODS) occurred in 12.8% (54/422) of patients in the PSS ≥ 2 group, whereas no cases of MODS were observed in the mild group (*P* < 0.001). These findings confirm that a classification of clinically significant poisoning (PSS ≥ 2) is strongly associated with severe, clinically objective organ injury and life-threatening systemic complications.

**Table 3:** Distribution of organ dysfunction and acute clinical complications between mild (PSS 0-1) and clinically significant (PSS ≥ 2) poisoning groups.

Acute Organ Dysfunction/Complications	Mild group (PSS 0-1) (*n* = 1,923)	Clinically significant group (PSS ≥ 2) (*n* = 422)	*P*-value
Acute respiratory failure, *n* (%)	2 (0.1)	112 (26.5)	<0.001
Toxic encephalopathy/coma, *n* (%)	0 (0.0)	95 (22.5)	<0.001
Acute liver injury, *n* (%)	15 (0.8)	84 (19.9)	<0.001
Myocardial injury, *n* (%)	10 (0.5)	76 (18.0)	<0.001
Acute kidney injury (AKI), *n* (%)	4 (0.2)	48 (11.4)	<0.001
Shock/circulatory failure, *n* (%)	1 (0.1)	35 (8.3)	<0.001
Multiple organ dysfunction syndrome (MODS), *n* (%)	0 (0.0)	54 (12.8)	<0.001

Values are expressed as counts (*n*) and percentages (%) within each group. PSS, Poisoning Severity Score; AKI, Acute Kidney Injury (defined by KDIGO criteria); MODS, Multiple Organ Dysfunction Syndrome (defined as simultaneous dysfunction of two or more organ systems). All *P*-values were calculated using Fisher's exact test.

## 4 Discussion

### 4.1 Main findings and epidemiological shifts

This 4-year, single-center retrospective cohort study comprising 2,345 pediatric cases provides a robust, contemporary, and highly granular analysis of the evolving landscape of acute pediatric poisoning. Our findings confirm a significant epidemiological shift in the post-pandemic era: while the overall incidence of acute poisoning presentations continues to climb, the toxicological spectrum has decisively transitioned from traditional agricultural chemicals toward pharmaceutical agents, most notably psychotropic drugs. Through rigorous multivariable logistic regression modeling, we successfully identified and quantified several independent risk factors driving clinically significant poisoning (PSS ≥ 2). Age over 12 years, intentional self-harm, a pre-hospital presentation delay exceeding 6 h, multiple-agent exposure, and exposure to high-lethality agricultural herbicides or psychotropic medications were established as robust predictors of clinical deterioration. Our model exhibited outstanding predictive performance (AUC-ROC = 0.892) and was thoroughly validated using bootstrapping. Importantly, the PSS ≥ 2 classification was strongly associated with patient-centered outcomes, including acute respiratory failure, toxic encephalopathy, and MODS. These findings may support early recognition and prioritization of high-risk pediatric poisoning cases, although prospective external validation is required before implementation as a formal triage tool.

### 4.2 Interpretation of trends: the adolescent mental health crisis

A prominent trend identified in our cohort is the significant increase in intentional self-poisoning utilizing psychotropic medications among adolescents (aged > 12 years) (17). This observation aligns with the reported global increase in adolescent mental health challenges, which have been exacerbated by the psychosocial reverberations of the COVID-19 pandemic (9). Prolonged social isolation, academic stress, disruption of normal developmental milestones, and increased screen time have collectively precipitated a surge in depressive and anxiety disorders among youth (18, 19). The widespread prescribing of antidepressants, sedatives, and atypical antipsychotics has consequently increased the availability of these agents within households, inadvertently creating a ready reservoir for impulsive self-harm (20, 21). The bimodal distribution captured in our heatmap (Figure 3) perfectly delineates this demographic dichotomy: toddlers predominantly engage in exploratory, low-lethality accidental ingestions of household products, whereas adolescents disproportionately present with calculated, high-dose intentional overdoses of psychotropics (20, 22). Our multivariable analysis underscores this reality, showing that age > 12 years (aOR 2.34), multiple-agent exposure (aOR 2.15), and intentional self-harm (aOR 3.12) are heavy independent drivers of clinically significant poisoning outcomes, which highlights the critical need to incorporate immediate psychiatric screening and crisis intervention into pediatric ED triage systems (23).

### 4.3 Mechanisms of severity and the role of extracorporeal interventions

The severity of poisoning in our cohort was heavily dictated by the specific mechanism of the toxicant and the presence of multiple ingested agents, which directly translates to the high rates of specific organ dysfunctions observed in our clinically significant cohort (Table 3). Poly-intoxication may result in additive or interaction-mediated toxicity beyond the expected effects of each individual agent, leading to more severe clinical presentations, lower levels of consciousness, clinically relevant drug-drug interactions, and increased requirements for mechanical ventilation, intensive care, and prolonged hospitalization, thereby substantially complicating clinical management (24). Furthermore, the pathophysiological mechanisms between agricultural and psychotropic exposures diverge drastically. Exposures to agricultural chemicals, particularly the bipyridylium herbicides paraquat and diquat, demonstrated the highest adjusted odds for adverse outcomes and accounted for all observed mortalities. The profound lethality of paraquat stems from its rapid accumulation in alveolar type I and II cells via the polyamine transport system, leading to cyclical redox reactions, devastating generation of reactive oxygen species (ROS), and ultimate pulmonary fibrosis (25). This pathophysiological pathway explains the high incidence of acute respiratory failure (26.5%) and MODS (12.8%) observed in our PSS ≥ 2 patients. Conversely, diquat primarily induces severe central nervous system toxicity, such as toxic encephalopathy, and acute kidney injury (26, 27). Our finding that toxic encephalopathy occurred in 22.5% of clinically significant cases and AKI in 11.4% aligns perfectly with these specific molecular mechanisms. Despite the low absolute frequency of these exposures in recent years due to regulatory bans, their case fatality rate remains disproportionately high.

In mitigating these severe outcomes, our study highlighted the extensive deployment of extracorporeal blood purification modalities, predominantly hemoperfusion (HP) and continuous renal replacement therapy (CRRT). While our observational design precludes direct causal assertions regarding the effectiveness of these interventions, the data highlight distinct clinical trajectories. Hemoperfusion, utilizing neutral macroporous resins or activated charcoal, has been used for selected highly protein-bound or lipid-soluble toxins, including some psychotropic drugs and specific agricultural agents (28). In the Sankey diagram, many clinically significant psychotropic-drug exposures that received blood purification were followed by survival, whereas deaths clustered among agricultural chemical exposures. Because treatment allocation was not randomized, this pattern should be interpreted as descriptive rather than evidence of treatment efficacy. This may partly reflect the pharmacokinetic profiles of selected psychotropic agents, including lipid solubility and protein binding, which may make selected agents more amenable to extracorporeal removal before extensive tissue distribution occurs. In contrast, the potential utility of extracorporeal clearance for paraquat/diquat is heavily time-dependent; a delay of over 6 h—which our regression model identified as a critical independent risk factor (aOR 2.78, 95% CI: 2.15–3.59)—may substantially reduce the potential benefit of extracorporeal clearance because of the rapid tissue distribution of these toxins (29, 30). From a resource-allocation perspective, blood purification should therefore be considered within a comprehensive clinical context, including toxicant type, estimated dose, time from exposure, clinical trajectory, and availability of critical care resources (31). Our internally validated model showed strong discrimination and may help clinicians identify patients who warrant early senior review and consideration of advanced supportive measures; however, external validation is needed before routine triage implementation.

### 4.4 Strengths and limitations

The principal strength of this study lies in its large sample size (*N* = 2,345) and its exclusive focus on the contemporary, post-pandemic timeframe (2021–2024), providing highly relevant data for current clinical practice. Furthermore, the rigorous utilization of the standardized PSS to distinctly separate mild from clinically significant cases allowed for a pristine multivariable regression analysis, identifying true independent prognosticators devoid of the “noise” created by benign ingestions. Additionally, the collection of detailed clinical organ-specific endpoints significantly strengthens the clinical utility of our findings. However, several limitations must be acknowledged. First, as a single-center retrospective study conducted in a major tertiary referral hospital, an inherent referral bias exists; our cohort likely represents a higher acuity population than community hospitals, potentially limiting the generalizability of the findings. Second, the reliance on electronic medical records makes the data susceptible to retrospective documentation errors, and the “time of ingestion” often relies on patient or caregiver report, introducing potential recall bias. Third, while we incorporated “pre-hospital interventions” into our model, it did not emerge as a significant protective factor. This is likely due to confounding by indication, wherein patients evaluated at local clinics and subsequently transferred to our tertiary center were inherently sicker. In addition, the pre-hospital intervention variable was binary and did not capture inter-hospital transfer status, exact timing or quality of decontamination, antidotal therapy, lipid emulsion therapy, or other toxin-specific interventions. Therefore, residual confounding related to pre-referral care cannot be excluded. Moreover, in multiple-agent exposures, assigning a dominant toxicant according to the predominant toxidrome may have introduced misclassification bias, particularly when several agents could plausibly contribute to the clinical presentation. Fourth, the PSS in our study reflected the worst clinical severity during the entire follow-up rather than just at initial ED presentation, which, while accurately reflecting ultimate severity, means the initial triage PSS might have been lower. Finally, toxicological screening (e.g., serum drug concentrations) was not universally available or standardized for all patients, necessitating reliance on clinical histories and syndromic diagnosis in a fraction of cases.

## 5 Conclusion

The landscape of pediatric acute poisoning has experienced a profound shift, defined by an alarming rise in adolescent intentional self-harm utilizing psychotropic drugs. Age over 12 years, suicidal intent, pre-hospital delays exceeding 6 h, multiple-agent exposure, and exposure to agricultural or psychotropic agents are independent risk factors driving clinically significant, life-threatening clinical outcomes. We validated that clinically significant poisoning classification (PSS ≥ 2) is strongly associated with objective patient-centered outcomes, including acute respiratory failure, toxic encephalopathy, and MODS. Our predictive model demonstrates outstanding performance (AUC-ROC = 0.892) and high stability. Recognizing these high-risk indicators is paramount. These indicators may help emergency clinicians prioritize early assessment and resource allocation for high-risk patients; however, whether model-guided triage or earlier advanced interventions can reduce morbidity and mortality requires prospective validation. Comprehensive socio-medical strategies, encompassing stringent chemical regulation and enhanced adolescent psychological support, are urgently required.

## Data Availability

The original contributions presented in the study are included in the article/Supplementary Material, further inquiries can be directed to the corresponding author.
